# Does the “Miracle Drug” of Environmental Governance Really Improve Air Quality? Evidence from China’s System of Central Environmental Protection Inspections

**DOI:** 10.3390/ijerph16050850

**Published:** 2019-03-08

**Authors:** Ruxin Wu, Piao Hu

**Affiliations:** School of Public Administration, Central South University, 932 Lushan South Road, Changsha 410083, China; piuu520@outlook.com

**Keywords:** central environmental protection inspections, air quality, campaign-style governance

## Abstract

Central environmental protection inspections have completed their goal of full coverage of 31 provinces in China, and more than 17,000 officials have been held accountable. The media has evaluated the effectiveness of central environmental protection inspections using the notions of “instant results” and the “miracle drug of environmental governance.” Can this approach effectively promote local environmental governance? This paper takes the treatment effect of central environmental protection inspections on air pollution as an example. Using the method of regression discontinuity, central environmental protection inspections are found to have a positive effect on the air quality index (AQI), but this effect is only short term and unsustainable. Additionally, there are inter-provincial differences. Judging from the research results on sub-contaminants, the treatment effect of central environmental protection inspections on air pollution is mainly reflected in PM_10_, PM_2.5_ and CO. Under the current situation in which PM_10_ and PM_2.5_ are the main assessment indexes, this phenomenon indicates that due to the political achievements and promotion of local officials and for reasons of accountability, it is more effective for the central government to conduct specific environmental assessments through local governments than to conduct central environmental protection inspections.

## 1. Introduction

In recent years, air pollution, represented by haze pollution, has affected a wide area of China. According to the Asian Development Bank, large cities in China with air quality that meets the criteria suggested by the WHO account for no more than 1% of the total [1]. Consequently, environmental protests such as the “Jiangsu Qidong Incident,” the “Zhejiang Ningbo PX Incident,” and the “BP Oil Spill” occur frequently. As the largest developing country in the world, China has received extensive attention regarding its contribution to and influence on global environmental change [2]. The control of environmental pollution has become a pressing policy issue facing the Chinese government [3]. Accordingly, 2019 is the year of the “battle to win the blue sky.” In 2018, the Chinese government continued to improve air quality to retain more blue sky for the masses, and it deployed a three-year action plan for the Blue Sky Defense War to significantly enhance the people’s “blue sky happiness” (on 27 June 2018, the State Council issued the “Three-Year Action Plan to Win the Blue Sky Defense War.” The Blue Sky Defense Three-Year Action Plan is a pollution prevention action plan deployed by the Chinese government to continuously improve air quality and retain more blue sky for the masses). These situations have led China to focus on environmental regulation as an effective approach to controlling pollution [4]. Constructing an authoritative supervisory and coordinating body is an important starting point for addressing the dilemma of environmental governance. Against this background, the environmental supervision system was developed.

The regional environmental management system has strong regional characteristics [5]. Before the establishment of the regional environmental supervision centers, the central government was responsible for the supervision and coordination of the overall environmental management system, and specific regional issues were resolved by local governments. However, the regional characteristics of regional environmental problem management conflict with the characteristics of environmental issues. The environmental responsibility of local governments is limited to administrative areas, and the lack of authoritative responsibility for cross-regional environmental issues has led to increased conflicts between local governments. Local governments lack the institutional capacity to effectively formulate and implement policies as well as the rationality of environmental spillovers across jurisdictions [6]. Therefore, the environmental spillover effects caused by local competition for economic resources provide legitimacy for the establishment of regional environmental agency. Establishing an authoritative supervisory and coordination mechanism helps to avoid the overall ecological problems caused by the deterioration of the transboundary environment [6]. In addition, China has a vast territory with differences in environmental conditions, resource endowments, economic development levels and population. There are significant differences in eco-environment sustainability and influencing factors. Environmental management issues vary from region to region, and it is difficult to adapt to uniform policies and standards [7]. Therefore, in the process of building a general environmental governance framework, the central government has become an important issue and challenge for the environmental governance of central and local governments. The system of regional environmental supervision centers is an administrative supervision system used by the state to strengthen the management of local environmental protection work, supervise outstanding problems, and break through the old administrative divisions to achieve cross-border solutions to environmental problems. However, since the establishment of the regional environmental supervision centers in 2002, it has failed to achieve its original goals, namely, to break local protectionism and mitigate environmental pollution. A strange phenomenon occurs in which the higher the demand for and expectations of a healthy environment, the worse the quality of the actual environment [8].

The key to a solution begins with an in-depth explanation of the reasons for the recent deterioration of environmental pollution in China. From this reality, we can see the embarrassing position of the regional environmental supervision centers within the entire environmental management system. This is why it does not work effectively. As an institution of the Ministry of Ecology and Environment, it is a bureau-level system. Although its administrative level is the same as that of other departments of the Ministry of Ecology and Environment, it is required to report to the Environmental Supervision Bureau, and that Bureau determines how to handle cases of environmental pollution [9]. The Ministry of Ecology and Environment also stipulates that the regional environmental supervision centers are forbidden from supervising the daily environmental protection work of the local government and from guiding the work of the local environmental protection department. the regional environmental supervision centers have functions with local governments and local environmental protection departments (Source: Former State Environmental Protection Administration, “General Administration Environmental Protection Supervision Center Formation Plan”, 8 July 2006. The State Environmental Protection Administration has now changed its name to the Ministry of Ecology and Environment.). It is difficult for the regional supervision center to accurately clarify the function of “not interfering with the work of local environmental protection agencies” and to fully perform the functions of regional supervision [10]. As a result, the Ministry of Ecology and Environment, the provincial government and the regional supervision center does not agree with the existing relationship model. The Ministry of Ecology and Environment believes that the regional environmental supervision centers have not played a good role in regional supervision and is limited in solving regional environmental problems. The regional environmental supervision centers do not have “organizational identity.” It is not considered an integral part of the Ministry of Ecology and Environment, and the organization faces a series of incentive problems. The provincial government believes that the regional environmental supervision centers is only the “eyes and ears” of the country—not the “problem solver” but rather the “problem maker” [6]. This unreasonable administrative-level setting has led to a very strange phenomenon: the organizational relationship that was originally parallel to the administrative structure has become the guidance for the organizational levels and supervision centers in practice. These restrictions have gradually made the regional environmental supervision centers a marginalized institution in the power chain and weakened their environmental governance effect. 

Theoretically, the “growth machine” theory describes cities as machines for increasing wealth, with politicians as the key operators. During the decision-making process, local officials always consider the interests of the merchants who support them, which also benefits them [11]. Chinese political and economic institutions are characterized as regionally decentralized authoritarian systems [12] in which the central government supervises personnel control over subnational governments while local governments control the bulk of China’s economy [13]. However, the appointment and promotion of local officials remain at the absolute discretion of higher-level government entities, which usually approve the political promotion of local officials based on an assessment in which economic performance plays a main role—the so-called “promotion tournament” [14,15]. This has led to strong incentives for local governments to promote economic development even at the expense of the natural environment, a situation called “pollution for promotion” [16]. Thus, local governments have great freedom in deciding how to implement policy [17]. These factors have led environmental problems to be the first to be eliminated from local government policy goals in every round of economic growth [18].

Conventional environmental protection mechanisms are difficult to effectively resolve given the deep-seated contradictions that characterize China’s system of environmental governance. To correct the old malpractices of the country’s environmental management system and to address the problems of environmental pollution that have long plagued China, the central government launched a strategy of central environmental protection inspections (see Appendix A ①) led by the Party Central Committee and the State Council. 

In 2015, the Party Central Committee and the State Council successively issued a series of important documents on the reform of China’s ecological civilization system (see Appendix A ②). Among these documents, the “Environmental Protection Inspection Program (Trial)” proposed the establishment of an environmental inspection mechanism to strictly address the responsibility for environmental protection. It is hoped that through the strong participation of the “central” authority, that is, in the name of the Party Central Committee and the State Council, local party committees and governments will examine their environmental protection duties and create strong pressure and behavioral obligations for leading cadres in political circles, forcing them to attach great importance to environmental protection and work to vigorously correct environmental violations. The Central Environmental Protection Inspector gives the environmental protection inspector a higher authority and “rigidity” in the name of the Party Central Committee and the State Council, emphasizing the “party and political responsibility” of environmental protection work, “double responsibility for one post,” and environmental protection of local party committees and governments.

According to the data in the 2016 National Haze City Rankings, seven of the top ten rankings are in Hebei. The air pollution control in Hebei has reached the point where environmental management cannot be delayed. At the end of 2015, the “first sword” of the environmental inspection team, headed by the central government, focused specifically on Hebei and conducted a pilot project of environmental protection inspections for one month. This team pursued strict investigation of and punishment for environmental pollution, chaos, dereliction of duty, misconduct, abuse of power, and other behaviors. Between 2016 and 2017, central environmental protection inspections fully investigated 31 provinces in China, with more than 17,000 officials held accountable.

The media evaluated the effectiveness of central environmental protection inspections against the notions of achieving “instant results” and dispensing the “miracle drug of environmental governance.” However, based on past experience regarding Chinese government’s repeated vows and growing investment in environmental protection, China’s environmental quality still appears to be continuously worsening, and the amounts of the main air pollutant and waste water emissions remain persistently high [2]. These observations lead to several questions. Are the central environmental inspections in China effective, and if so, to what extent? Is this truly an effective approach to promoting local environmental governance? After the inspection team leaves, is the situation in the inspected area rectified? Is this type of campaign-style governance (see Appendix A ③) a passing storm or a long-term mechanism?

To answer the above questions, it is necessary to scientifically and effectively evaluate the implementation effect of central environmental protection inspections. This paper focuses on AQI and uses regression discontinuity to evaluate the impact of the implementation of the central environmental protection inspections on AQI. This paper also analyzes differences in the governance capacities of the inspected provinces and the governance effects of the other six sub-contaminants in the evaluation system. Because central environmental protection has constituted a major innovation in China’s environmental regulatory system since the 18th National Congress, research on this innovation can enrich theoretical research on the supervision system; furthermore, it can provide empirical evidence to inform reforms of China’s environmental supervision system and to promote the development of central environmental protection inspections. Thus, this research has important theoretical and practical significance.

Our findings contribute to the literature in two ways. First, the present study adds new empirical evidence to the public management literature on the governance behavior of local government officials. The public management literature has long attempted to understand the effect of local government on implementing certain policies. Our results suggest that in the face of inspections, local governments choose campaign-style governance for their own interests to achieve relevant environmental goals. The target-based evaluation system can be effective in motivating bureaucrats to shift their efforts toward tasks that are more heavily weighted by the evaluators and seek to achieve the targeted policy output. 

Second, the present study addresses an important yet relatively neglected topic in the environmental literature – the empirical relationship between central environmental inspections and environment governance. The empirical literature on the relationship between environmental inspections and environmental inspections is rather limited. Existing studies have usually focused on estimating the relationship between environmental and economic policies [19]. The agency of the central environmental protection inspector is an emerging system, and its effectiveness has not yet been verified from an empirical perspective. Our findings contribute to the small but growing literature on how strongly central governments can affect the adoption of more stringent environmental inspections policies.

The remainder of the paper proceeds as follows: Section 2 is a literature review of the central environmental protection inspections and regression discontinuity. Section 3 outlines the methodology and data used in this paper. Section 4 assesses the implementation of central environmental protection inspections on pollutants and provinces. Section 5 discusses the empirical results. Section 6 concludes the paper and provides some policy suggestions.

## 2. Related Literature

Although there has been a growing number of literature reports on the impact of economic development on the environment, very few studies have investigated the effectiveness of environmental inspections [2]. Some researchers have focused on improving the environmental governance effect of the environmental protection inspection system, aiming to understand the effectiveness of China’s environmental governance and environmental reform by exploring the relationship between the two. Before the implementation of central environmental protection inspections, the regional environmental supervision centers were the main body that implemented daily supervision work, and the environmental supervision bureau was in charge of contact and business guidance [20]. Mao and Luo [6] believe that the contradictions and conflicts between nationalism and regionalism in the current regional supervision have made it difficult for the regional supervision centers to play their expected role of “supervising and checking” in a practical sense. Fang [21] noted that the establishment of China’s regional environmental protection inspection center is a top-level attempt to address the problem of local environmental governance, but its status, financial support, work objects, operational efficiency, and internal construction all have corresponding loopholes. Fang thus called for legislation to resolve the ills of the regional environmental protection inspection system. Chen [22] noted that based on the operational practices of the six regional environmental supervision centers, the system has failed to achieve the original intention of breaking up local protectionism and representing the state in order to supervise the local implementation of environmental responsibility. Instead, it has increasingly become the responsibility of the office of the Ministry of Ecology and Environment to handle temporary and local affairs. 

**Figure 1:**
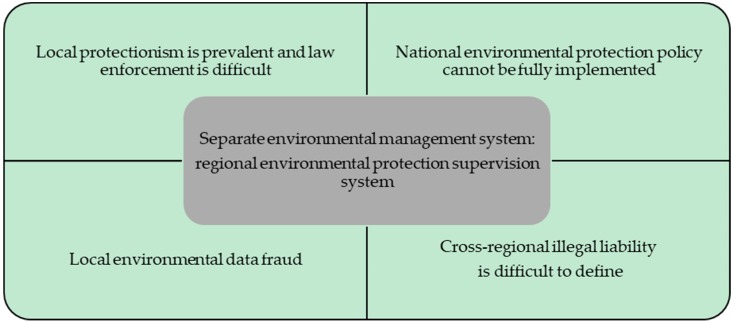
Problems in the regional environmental protection supervision system.

Before the implementation of the central environmental protection inspection system, most research focused on the regional environmental protection supervision system. Judging from the existing research, the actual implementation of the regional environmental supervision centers has not achieved significant results (Figure 1). The subsequent central environmental protection inspections are a major reform of China’s environmental supervision system. Zhang [23] found that under the party and government system, the effect of giving political incentives to the party secretary is more obvious than the effect of incentivizing the mayor, and the party committee secretary may play a stronger role in environmental governance. The new central environmental protection inspections are based on the same characteristics of the party and government, and the main features are different from the previous approach to supervision [24]. Liu et al. [25] noted that through central environmental protection inspections, local party committees and governments at all levels have further implemented the environmental protection responsibilities of both the party and government, documents on the long-term mechanism of environmental protection have been promulgated, and a large number of prominent environmental issues affecting the masses have been resolved. The intensity of the inspections is unprecedented, as is the level of problem solving. Feng [26] noted that the central environmental protection inspection system is the embodiment of the central environmental protection inspector’s pollution control. It is also a warning to the local government, which can greatly enhance the red-line awareness of local environmental protection and accelerate the transformation and upgrading of local industries.

However, scholars have expressed doubts about the effectiveness of central environmental protection inspections. For example, Lo [27] mentioned that the complexity of the governance structure and the differences among local governments pose serious challenges to the central government’s collection of information. It is difficult for the central government’s inspection assessment to cover all aspects of environmental protection, and some assessments can only be based on local government self-assessment reports. This situation gives local governments the opportunity to provide false reports. Chen [22] puts China’s environmental protection supervision system in the context of historical development and analyzes the evolutionary path from “supervising enterprises” to “supervising government” to “party and government responsibilities,” noting that the current system belongs to the “campaign-style governance” model. Central environmental protection inspections cannot ensure the sustainability of governance and the sustainability of institutional operations. Chen further notes that the rule of law should be used to resolve the shortcomings of “campaign-style governance.” Chen [28] uses case studies to demonstrate that the supervision mechanism cannot fully realize the integration of bureaucratic governance and campaign-style governance from the perspective of operational effectiveness. In all types of supervision and inspection, there is always information concealment, inspection fraud, data fabrication, and so on. The problem is that bureaucratic organizations cannot be completely penetrated by the will of the leadership. Shi et al. [29] used the regression discontinuity method to evaluate the effect of this system on air pollution in 25 cities that made environmental protection inquiries. Studies have shown that local governments use “coping” behaviors to cover up the truth. Moreover, the arranged inquiries of the Ministry of Ecology and Environment, which is based on campaign-style governance, have only a very short-term effect on air pollution control.

In empirical research, regression discontinuity has been used to evaluate the effectiveness of environmental policy. Almond et al. [30] used a regression discontinuity design to study the impact of China’s heating policy on local environmental pollution. During the 1950–1980 period of central planning, due to budgetary limitations, only the Huai River and the area north of the Qinling Mountains were able to receive free coal heating. Using a unique data file on the air pollution concentration in 76 Chinese cities, the heating policy led to dramatically higher levels of Total Suspended Particulates (TSP) in North China, and the TSP levels in North China are 5–8 times those of the United States. Tang and Liu [31] used the method of regression discontinuity design to test whether the mandatory target system (MTS) has improved the environmental governance performance of local governments in China. The results of that research demonstrate that the MTS has had a positive effect on environmental performance; however, the regression discontinuity design illustrates that the reward and punishment measures in the MTS have had no significant effects on provincial environmental performance. Zhang et al. [32] used fuzzy regression discontinuity to study whether China’s central supervision and local environmental decentralization management could be combined. The results suggested that central supervision significantly reduced industrial COD emissions by at least 26.8% and that the effect gradually increased with time, thus providing strong evidence supporting the central vertical regulatory reform.

The academic community has two different views on the effectiveness of central environmental protection inspections. One view is that central environmental protection inspections have achieved a breakthrough effect that has greatly improved the environment. Some scholars feel that central environmental protection inspection is the same as the previous system of campaign-style governance; it has a certain effect but not a long-term one. However, the controversial estimation results indicate that neither of the two views has been fully confirmed. How should we view these differences in the understanding of the effectiveness of central environmental protection inspections? In fact, theoretical disputes precisely reflect the multifaceted nature of reality. Central environmental protection inspections have only just begun. Determining their specific effects will require many demonstration tests. Based on the existing literature, the current discussion of central environmental protection inspections focuses on media reports and government departments of information disclosure. Most of the literature follows the three-stage theoretical thinking of “problems–causes–suggestions.” Research on central environmental protection inspections is only discussed in specific cases. Few documents have studied the implementation effect of central environmental protection inspections from a strictly empirical perspective. In the current context of China’s current environmental supervision failure, exploring the governance effect of the central environmental protection inspection system is conducive to identifying its defects and has important practical significance for reforming and improving the environmental management system and accumulating environmental supervision experience. Therefore, this paper is based on a regression discontinuity design and uses the central environmental protection inspection reports to examine air quality data to analyze the effect of central environmental protection inspections on the AQI and various sub-contaminants and to understand the differences in the governance capabilities of the provinces under central environmental protection inspections.

## 3. Methodology and Data

### 3.1. Regression Discontinuity Design Model

This paper uses the method of regression discontinuity design [33] to assess the environmental governance effects of central environmental protection inspections. Regression discontinuity design has been widely used in air pollution literature, such as using time as a breakpoint to investigate whether air quality mutated before and after an event [34,35,36]. In the evaluation of the central environmental protection inspections, if it can be observed that air quality changes abruptly before and after the implementation of central environmental protection inspections and if other influencing factors can be identified as continuous changes, there would be reason to believe that this air quality mutation is brought about by the implementation of central environmental protection inspections. That is, there would be reason to believe that central environmental protection inspections have improved air quality. The regression discontinuity design model [33] of this paper is designed as an equation:

(1) $$AQI_{cd}=\beta_{0}+\beta_{1}inspection_{cd}+\beta_{2}f\left(x\right)+\beta_{3}inspection_{cd}f\left(x\right)+\alpha X_{cd}+\delta_{c}+\mu_{d}+\varepsilon_{cd}$$

where c is the province, d is the date, $AQI_{cd}$ is the air quality index of province c on date d, $inspection_{cd}$ is representative of the dummy variable of the central environmental protection inspections, and c province is 1 after the date d of the inspections or 0 before. x is an execution variable that indicates the number of days from the date of the central environmental protection inspections. It is 0 on the day of the inspection, greater than 0 after the inspection, and less than 0 before the inspection, and f(x) is a polynomial function with x as the independent variable. $X_{cd}$ is a set of weather control variables, including maximum temperature, minimum temperature, whether there is rain, whether there is snow, and wind size variables to control the impact of weather factors on air quality. $\delta_{c}$ is the regional fixed effect for the city c. $\mu_{d}$ is a time-fixed effect. This article controls for the year, month, week, and legal holidays, etc., to control the seasonal factors and the impact of people’s working schedule on air quality. $\varepsilon_{cd}$ is a stochastic disturbance term. In equation (1), the main concern of this paper is $\beta_{1}$, which captures the difference in air quality before and after the central environmental protection inspections. Of course, many factors affect the air quality of a city, such as geographical factors, industrial structure, energy structure, and car ownership, but these urban characteristics generally do not change in the short term (the time span of the data in this paper is 5 months). Thus, these factors have been controlled by using the fixed effect model of panel data, and the missing variable bias has been alleviated to some extent [36].

### 3.2. Data Description

**Figure 2:**
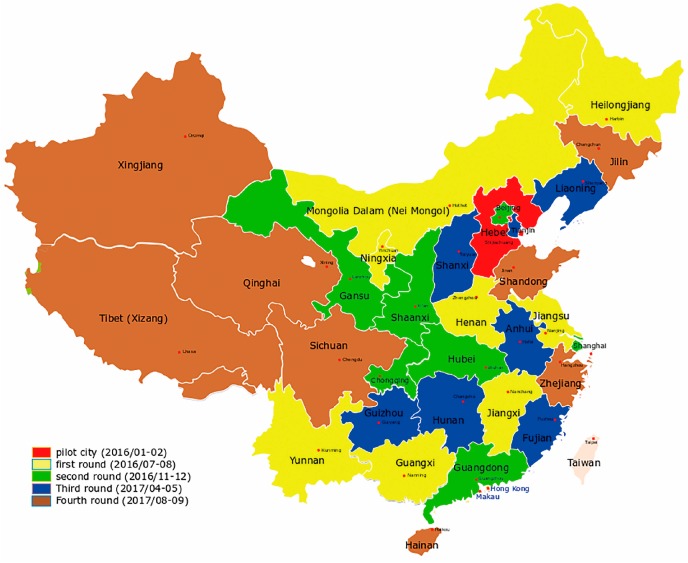
The Central Environmental Protection Inspectorate achieved full coverage in 31 districts in batches.

At the end of 2015, the “first sword” of the environmental inspection team, headed by the central government, focused specifically on Hebei and conducted a pilot project of environmental protection inspections for one month. Between 2016 and 2017, central environmental protection inspections fully investigated 31 provinces in China (Figure 2).

The AQI value is obtained by indexing the concentrations of various individual pollutants and calculating these sub-indexes. The classification calculation used by AQI is based on the Air Quality Standard (GB3095-2012). There are six types of pollutants in the evaluation system: SO_2_, PM _2.5_, PM _10_, O _3_, CO and NO _2_. The air quality data used in this paper are based on the monitoring data of AQI, PM_2.5_, PM_10_, S0_2_, N0_2_, O_3_, CO, temperature, wind level and wind direction provided by the China Air Quality Online Monitoring and Analysis Platform (Source: China Air Quality Online Monitoring and Analysis Platform, https://www.aqistudy.cn/historydata/). 

The mean difference between the explained variable and other control variables before and after the cut point is shown in Table A1 (see Appendix B, Table A1). AQI, PM_2.5_, PM_10_, CO and SO_2_ were lower by approximately 10% after the central environmental protection inspections. On average, the temperature increased significantly after the implementation of central environmental protection inspections. During this period, there was no clear change in rainfall. The occurrence of snow days was reduced to a small extent, and the difference was significant at the 1% level. 

These common factors and other unobserved factors together determine the significant decline in AQI, PM_10_, PM_2.5_, CO, NO_2_, and SO_2_. Therefore, it can be speculated that the changes in the interpreted variables after the implementation of central environmental protection inspections are not only due to changes in the implementation of the inspection but also may benefit from favorable meteorological conditions and the effects of seasonal changes. Therefore, it is necessary to control meteorological factors to identify the causal effects of central environmental protection inspections on air pollution improvement and the effects of seasonal changes.

**Figure 3:**
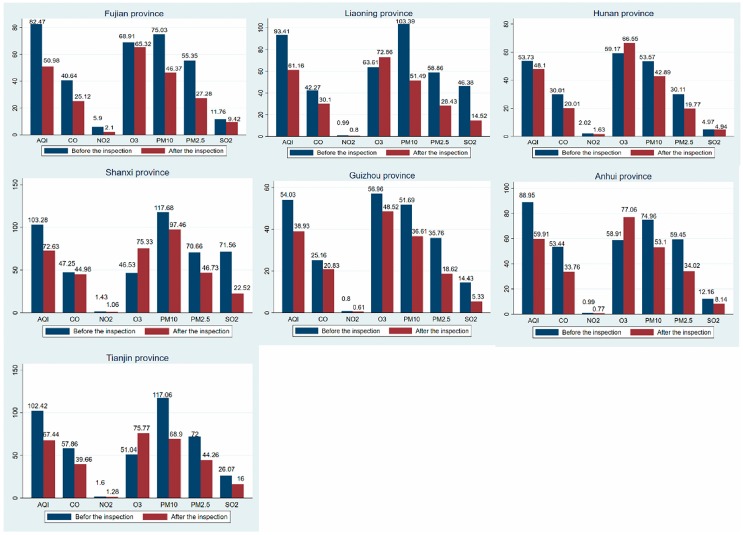
Air quality difference map before and after inspections.

Figure 3 shows the mean difference in air quality before and after the third round (taking into account the length of the article, this section shows the third round of environmental inspections) of central environmental protection inspections. Except for O_3,_ the concentrations of other pollutants in the seven provinces showed different degrees of decline. The improvement in PM_2.5_ and PM_10_ in each province was very large. In addition to the relatively small improvement in the AQI index in Hunan and Guizhou, other provinces have seen significant improvements. In trying to explain these changes, it can be seen that the provinces with greatly improved air quality originally had poor air quality, and the room to improve was therefore greater than that of Hunan and Guizhou, where the air quality was better.

## 4. Empirical Analysis

### 4.1. Regression Discontinuity Design

**Figure 4:**
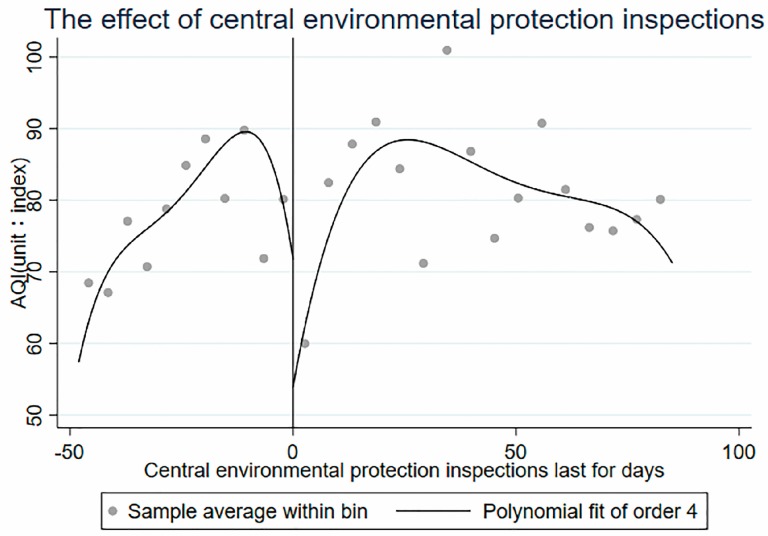
Contaminant concentration: order-4 polynomial fitting curve.

In this paper, using the Stata software (StataCorp LLC, Lakeway, Texas, USA), the scatter plot near the cut point and its fitting curve are plotted with the command rdplot, as shown in Figure 4. The quartic function fits well with the change in pollutants before and after the implementation of central environmental protection inspections, and the AQI has a cut point near the implementation of central environmental protection inspections. Therefore, this paper can further use the regression discontinuity to estimate the local average treatment effects (LATE) at the cut point. A study by Lee and Lemieux [33] shows that to achieve consistent estimates of regression discontinuity, control variables are not required, and these additional control variables are added only to improve efficiency. To reduce the residual variance and improve the efficiency, as described by Sun [37] and Shi [38], the factors affecting air quality are controlled accordingly.

Taking into account the interactions of polynomials, the regression results for one to four item functions are listed separately. Table 1 shows the regression discontinuity results of AQI. From the second line in Table 1, it can be seen that central environmental protection inspections have a negative impact on the air quality index from linear to quartic polynomial (that is, the central environmental protection inspections have a positive impact on air quality), which indicates that central environmental protection inspections have an optimization effect on the improvement of air quality to some extent.

Previous studies have shown that bandwidth may also affect the robustness of the results of the regression discontinuity estimation. Therefore, we use the 10, 15 and 20 days and 30 days before and after the environmental inspection as the bandwidth for our robustness analysis. The estimated results are shown in Table 2, which shows that the air quality has improved to some extent. The regression results of different bandwidths support the empirical conclusions of Table 1, which is consistent with the above conclusions, thus demonstrating that the conclusions of this paper are very robust to different bandwidths.

According to the results of the regression discontinuity above, central environmental protection inspections have a certain air governance effect. The question is whether the blue sky created by central environmental protection inspections is sustainable. What is the rectification situation of the inspected area after the inspection team leaves? Is there a situation where “the inspection team is gone and the pollution is back?” Shi et al. [38] took the local “two sessions” as their research object and found that although local governments increase environmental protection during politically sensitive periods and reduce the level of air pollution during those times, the blue sky brought about by such measures is temporary. The improvement is not sustainable, and the level of smog will soon return to normal. Sun et al. [39] studied APEC Blue and found that in just 10 days, the “APEC Blue” that was based on campaign-style governance improved the haze that would have otherwise taken 20–30 years to resolve. However, the effect of “APEC Blue” disappeared after the meeting. Therefore, to investigate whether the blue sky created by central environmental protection inspections is also a short-term phenomenon, this paper adds dummy variables of different time periods after central environmental protection inspections and sets the virtual days to 5 days, 15 days, 30 days, 40 days and 60 days after the inspection. These dummy variables are put into the regression equation at the same time. The regression results are shown in Table 3. Although the effect of central environmental protection inspections is still negative, the significance of the last several periods is not significant. This indicates that central environmental protection inspections may have only short-term effects that are not sustainable.

### 4.2. Other Sub-Contaminant Regression Results

To further discuss the impact of central environmental protection inspections on the synthesis of individual pollutants in AQI, this paper returns the average daily value of the six individual pollutant concentrations as the explanatory variables. The regression results are shown in Table 4. Central environmental protection inspections only have a governance effect on PM_10_, PM_2.5_ and CO. The coefficient regression results of other single pollutants are very small and have not reached a significant level. However, NO_2_ has increased. Central environmental protection inspections have had a significant effect on PM_10_, PM_2.5_ and CO but not on other individual pollutants. NO_2_ even showed an increase during the inspection period.

### 4.3. Differences in Governance Effects of Inspected Provinces

To further understand the environmental governance capabilities of each inspected province and to identify pollution control priorities for each region, a regression was performed with 7 pollutants from each province as explanatory variables.

**Figure 5:**
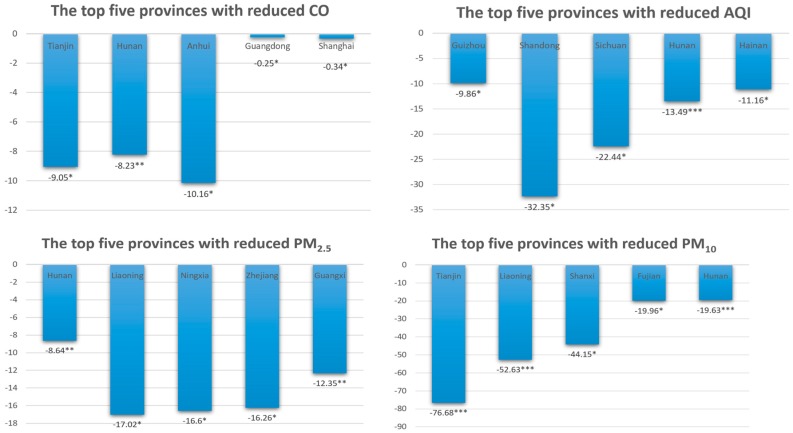
Ranking of pollutant reduction in each province.

From Table A2 (see Appendix B, Table A2) and Figure 5, we can see that the governance results for PM_10_ and AQI in each province are very significant. Other pollutants have different governance effects depending on the province. However, even in provinces in the same region, the effects of pollutants may have different effects (for example, Qinghai, Gansu and Tibet all belong to the western region (China’s regional differences can be roughly divided into three economic zones—the eastern, central and western regions—based on characteristics such as development level, geographical location, economic linkages, and energy consumption structure) but the governance effects of CO and NO_2_ show opposite results). The differences in these results may be related to the geographical factors, economic base, industrial structure, technical conditions and pollution prevention efforts of the provinces. The chain effect caused by this series of factors is the difference in local government environmental governance behavior. This explains why provinces in the same region have different governance effects on different pollutants. Under the impetus of central environmental protection inspections, we can see that the ranking of pollutant reduction in Hunan Province is very high, which may be related to the fact that the number of accountants is the highest among environmental protection inspectors. Other provinces with more pollution reductions were originally the areas that were most affected by air pollution. Therefore, the range that can be adjusted is large.

### 4.4. Validity Test of RDD Results

#### 4.4.1. Drive Variables Are Not Manipulated by Humans

The benchmark empirical results obtained above demonstrate that central environmental protection inspections have certain air pollution control effects. However, because the validity of the results of the regression discontinuity may also be affected by other conditions, this section will conduct a robustness test.

First, it is necessary to detect whether the individual can accurately manipulate the cut point. The test method is to judge whether there is a jump in the distribution of the forcing variable at the cut point. Using the McCrary [40] test to test the drive variable (the inspection date), we find θ = 1.29, and the standard error is 0.18 less than 1.96, so the acceptable forcing variable passed the McCrary test; in other words, there is no manipulation phenomenon on the inspection date. In fact, because the date of the inspection is determined by the central government and then shared with the local government, it is difficult for the local government to manipulate the day when the inspection will occur.

#### 4.4.2. Smoothness Assumption

**Figure 6:**
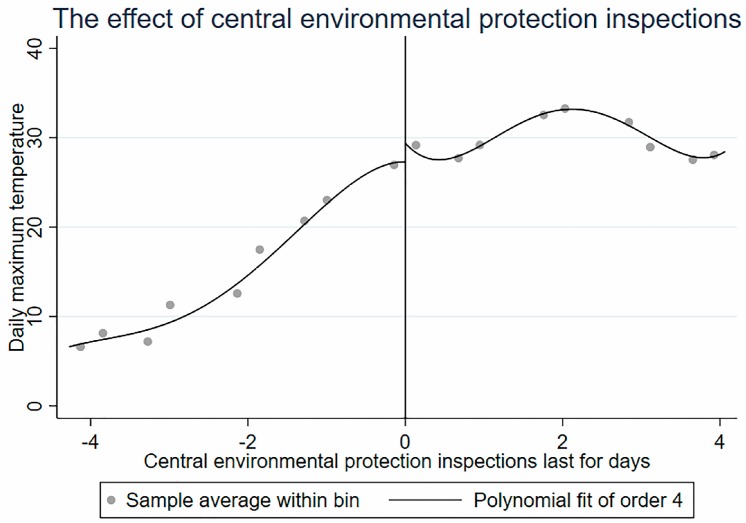
Smoothness hypothesis test chart.

The smoothness hypothesis is that other covariates affecting the results should not have obvious jumps at the cut point. The meteorological conditions, such as the highest temperature, the lowest temperature, whether there is rain, whether there is snow, whether it is a holiday, and the wind speed, are confounding factors affecting air quality. The partial regression discontinuity graph of these variables relative to the reference variable is shown in Figure 4. There is no obvious jump at the cut point. Using each covariate as a result variable and selecting a fourth-order polynomial for regression discontinuity, the coefficient of the cut point is very small and not significant, so the covariate can be considered to be continuous at the cut point (See Figure 6). Other confounding factors that have an impact on air quality have had little impact on the air quality control effects of central environmental protection inspections. The change in air quality is entirely caused by central environmental protection inspections.

## 5. Discussion

Why do central environmental protection inspections only have short-term effects that are not sustainable? The answer mainly lies in the fact that central environmental protection inspectors belong to the category of campaign-style governance. The political blue sky not only emerges during international events but has also become a routine vanity project for environmental pollution control [13]. Central environmental protection inspections are characterized by many different activities, such as manufacturing public opinion, comprehensive mobilization, layer-by-layer transmission pressure, mobilizing the resources of all parties, personal deployment of high-level political leaders of the central government, participation of senior officials at the provincial and ministerial levels, and media portrayal of party and state leaders. These activities all reflect the government operation mode of “campaign-style governance,” which enables the department to maximize cooperation in a short period of time based on various resources and functions [41]. However, this “task-driven” model of multiple goals is likely to shape local government roles and create functional distortions [42]. Furthermore, “campaign-style governance” is endogenous to the centralized system, and its processes, characteristics, and effects are similar to the “stimulant effect.” This situation stems from the fact that the authoritarians, through their persistent anxiety, generate excitement; the process usually starts suddenly, and then the action escalates and finally stops. The goal can be achieved to a certain extent, but it cannot eradicate the conventional crisis of governance. After the initial upheaval, the role of stimuli disappears, and everything remains the same [43]. Because the root cause of the incident is difficult to address through “campaign-style governance,” after the emergency management phase is over, the reason for the initial incentive will rebound and the problem will become more serious. In the next emergency treatment, the government will have to pay more costs [44]. These characteristics show that the central environmental protection inspection system, as a form of “campaign-style governance,” achieves short-term effects, but it cannot guarantee the sustainability of governance effects and the sustainability of institutional operations. Although air quality may improve substantially in a short period of time, it is not sustainable.

From the perspective of the treatment effect of sub-contaminants, central environmental protection inspections have a significant effect on PM_10_, PM_2.5_ and CO but no significant impact on other individual pollutants. This is because people are more sensitive to fog and haze than to other aspects of air pollution. The central government uses PM_2.5_ and PM_10_ as the main assessment basis for air pollution control (according to the Notice of the “Measures for the Implementation of the Air Pollution Prevention and Control Action Plan (Trial)” issued by the General Office of the State Council in April 2014, Beijing-Tianjin-Hebei and the surrounding areas, the Yangtze River Delta region, the Pearl River Delta region and Chongqing Municipality use PM_2.5_ as an indicator. The proportion of the five-year average concentration decreased as an indicator, whereas in other regions the annual average concentration of PM_10_ decreased as an indicator). Affected by the pressure of performance appraisal, local governments become enthusiastic about strengthening their governance. The assessment of the local government by the central government may be more obvious than the promotion effect of the central environmental protection inspection system with its punitive effects. The performance evaluation system caused a significant decrease in PM_10_ and PM_2.5_ emissions. In particular, our results suggest that a target-based evaluation system can be effective in motivating bureaucrats to shift their efforts toward tasks that are more heavily weighted by the evaluators and to seek to achieve the targeted policy output. However, although environmental protection is officially and practically incorporated into the cadre performance evaluation, different pollution types are treated differently. Liang and Lang [45] revealed that when air pollution is more tangible, such as PM_10_ and PM_2.5_ particulates that are directly visible to the public, more effort is made to control it. Indices for less tangible pollution, such as water pollution and other contaminants that are not included in the performance assessment, are not properly addressed. This hypothesis is consistent with the conclusions reached in Tang and Liu [31] and Liang and Lang [45]. On the other hand, 40% of PM_2.5_ comes from coal. At present, coal accounts for 64% of China’s energy structure. This dependence on coal makes PM_2.5_ treatment extremely difficult, which is also an important reason why the inspector effect of PM_2.5_ is smaller than that of PM_10._

In addition, in conducting central environmental protection inspections, provinces are heterogeneous, and the way different provinces approach these inspections may depend on their opportunities to obtain benefits from environmental governance and their ability to control environmental pollution [46]. The causes of atmospheric pollution are complex and closely related to the geographical natural factors, local industrial structure, and energy consumption habits, and they have obvious regional characteristics. Due to China’s vast territory and its serious imbalances in regional development, different regions have significant heterogeneity in terms of their economic base, industrial structure, technical conditions, and energy structure. The ability to prevent air pollution, as well as the technology and management capacity to do so, must coexist alongside large geographical differences [47].

## 6. Conclusions

This article presents empirical research, conducted using regression discontinuity, on the central environmental protection inspections. Central environmental protection inspections were found to have a positive effect on the improvement of the air quality index to a certain extent. However, the empirical results in this paper indicate that, in general, the effectiveness of central environmental inspections is rather limited. Among the six pollutant emissions, only PM_10_, PM_2.5_ and CO emissions were effectively reduced by the environmental inspections, which is in line with the central government’s assessment of the main pollutants. For the remaining three pollutants (i.e., O_3_, NO_2_ and SO_2_) the environmental inspections did not significantly lead to lower emissions or discharge. In most cases, the current implementation of China’s environmental inspections did not meet the expected results of the effective containment of pollutant emissions, and the current central-to-local assessment system may be more powerful than the central environmental protection inspection agency’s governance of air quality.

At the same time, based on the regression results of the provinces in the third round of inspections, the air pollution control effect in Hunan Province is the most obvious. These findings show that there are certain differences in the governance capabilities of the provinces under central environmental protection inspections. In addition, the empirical research in this paper found that the effect of central environmental supervision on the treatment effect of air quality is unsustainable. A significant effect is only found in the short term; that is, the central environmental inspector engages in campaign-style governance, and the effect of such governance is rapid but not sustainable. Central environmental protection inspections promote the environmental governance of local governments by means of political mobilization and adopt top-down supervision to ensure the strict implementation of environmental policies. This may lead to problems such as “surface management,” “pretend governance,” and “perfume governance” to handle the central government (Source: the people’s daily of china, http://politics.people.com.cn/n1/2018/0816/c1001-30231207.html).

In 2018, the Central Environmental Protection Inspector began the first round of “looking back.” (see Appendix A ④) In the process of “looking back,” the issue of rectification was continuously exposed. Before “looking back,” the “rectified” report submitted by the local government created a loophole when it looked back again. Through “reformation on the surface,” “reformation on paper,” and “formalism,” the localities have responded to the inspection group in various ways, which also illustrates the deepening of environmental pollution and the difficulty of promoting local environmental pollution control work. Under these circumstances, increasing the governance effect of central environmental protection inspections has become a top priority.

### Policy Suggestions

(1) It is recommended that the results of central environmental protection inspections be incorporated into the results of local government performance appraisals to give the former greater weight and link them with the official performance of officials.
(2) Due to the differences in governance capacity and different environmental conditions in each province, air pollution control needs to be adapted to local conditions; in other words, local governments are required to adopt different governance measures according to the industrial structure, energy consumption and economic development of each province [47]. Local governments should formulate corresponding rules and regulations, refine relevant environmental laws and regulations, adapt to local conditions, and formulate reasonable discharge standards according to the specific conditions of the region, such as economic development status, pollutant discharge ratio, and treatment technology level. The central government should adopt different environmental control measures and special inspection methods according to the actual situation of each province instead of all provinces adopting a “one size fits all” approach to governance. Central environmental protection inspections are an effort of the central government to understand local environmental governance. Inspection is not only a form of punishment and accountability but also a form of governance. Therefore, after the inspection, the provinces with more governance challenges should be given greater financial and time support, and the provinces with weak governance should be given more technical assistance. Accountability and punishment are only a means to an end, and the genuine improvement of environmental governance is the real purpose of central environmental protection inspections.
(3) Environmental governance is a protracted war. It requires a large amount of investment in people and property. The districts that have completed the rectification tasks of environmental protection inspectors and achieved outstanding effects via rectification have actively helped to obtain the central government’s environmental protection funds and increased the subsidies for environmental protection funds.
(4) Because the pressure of central environmental protection inspections has a certain effect in the short term, the environmental governance effect will gradually weaken after the inspection team leaves, and the local government will conduct mobile governance based on the results of the inspection. To consolidate the results of central environmental protection inspections, a strict long-term mechanism for central environmental protection inspection should be established to put the “environmental storm” under the control of the system. In the broadest sense, discussions about climate change by ‘ecoelites’ in China take place almost exclusively within technocratic and regulatory discourses that make little or no mention of society [48]. Therefore, we should unblock the channels of interest expression of multiple subjects, fully mobilizing the role of civil society and NGOs, and the mainstream media should be used to shape the power of public opinion [49] to regulate and guide the environmental governance behavior of local governments. In addition, the environmental supervision of the National People’s Congress and the CPPCC should be strengthened, and the multiagent supervision and management system should be further improved to internalize environmental governance into the daily behavior of local governments.

Central environmental protection inspections cannot become merely gusts of wind. It is necessary to actively summarize and combine experience, explore more effective environmental monitoring functions, and promote environmental governance and protection to a new level.

## Figures and Tables

**Table 1:** AQI regression results.

Variable Name	Linear Polynomial	Quadratic Polynomial	Cubic Polynomial	Quartic Polynomial
Central Environmental Protection Inspections	−8.89 ***	−9.09 **	−11.73 ***	−12.96 ***
Rain or not	−11.43 ***	−11.434 ***	−11.48 ***	−11.51 ***
Snow or not	1.84	1.85	1.52	1.48
Working day or not	2.87 *	2.87 *	2.75	2.76
Daily maximum temperature	0.29	0.29	0.30	0.29
Daily minimum temperature	−0.92 ***	−0.91 ***	−0.85 **	−0.81 **
Season	−7.49 ***	−7.56 ***	−7.43 ***	−7.44 ***
Wind power	−1.78 ***	−1.78 ***	−1.76 ***	−1.76 ***
cons	102.66 ***	102.87 ***	101.05 ***	101.49 ***

Note: * Denotes *p* < 0.10. ** Denotes *p* < 0.05. *** Denotes *p* < 0.01. The regression also includes weather variables, regional fixed effects, and time-fixed effects such as year, month, week, and holiday.

**Table 2:** Results of bandwidth sensitivity.

Bandwidth	±10	±15	±20	±30
Central environmental protection inspections	−8.037 *	−13.576 ***	−18.439 ***	−20.203 ***
	(–2.32)	(–4.78)	(–7.91)	(–9.78)
N	4510	4510	4510	4510

Note: * Denotes *p* < 0.10. *** Denotes *p* < 0.01. The regression also includes weather variables, regional fixed effects, and time-fixed effects, such as year, month, week, and holiday.

**Table 3:** Regression results for multiple periods after the inspection.

Variable Name	5 Day	15 Day	30 Day	40 Day	60 Day
Central environmental protection inspections	−8.89 ***	−10.961 ***	−9.186 ***	−10.986	−9.050
cons	102.66 ***	101.811 ***	92.555 ***	100.788 ***	103.522 ***

Note: *** Denotes *p* < 0.01. The regression also includes weather variables, regional fixed effects, and time-fixed effects, such as year, month, week, and holiday.

**Table 4:** Regression results of other sub-contaminants.

Full Sample	PM_2.5_	PM_10_	CO	NO_2_	SO_2_	O_3_
Central environmental protection inspections	−8.46 ***	−20.62 ***	−5.68 ***	2.071 *	0.819	−8.976
Rain or not	−5.82 ***	−10.95 ***	1.15 **	−3.29 ***	−2.86 ***	−8.464 **
Snow or not	0.82	−6.07	−0.98	−1.75	1.24	−3.42
Daily maximum temperature	−0.58 *	0.92 ***	0.68 ***	−0.13	0.14 *	2.671 ***
Daily minimum temperature	−0.27	−1.23 ***	−0.98 ***	−0.08	−0.91 ***	−0.88
Holiday virtual	YES	YES	YES	YES	YES	YES
Season virtual	YES	YES	YES	YES	YES	YES
Fixed effect	YES	YES	YES	YES	YES	YES
N	4435	4435	4435	4435	4435	4435
R^2^	0.1	0.07	0.2	0.11	0.17	0.21

Note: * Denotes *p* < 0.10. ** Denotes *p* < 0.05. *** Denotes *p* < 0.01. The regression also includes weather variables, regional fixed effects, and time-fixed effects such as year, month, week, and holiday.

## Appendix A

① Central environmental protection inspections: Accountability is the top priority of the central environmental protection inspections. The focus of the inspection is on the provincial party committee, the government and relevant departments, and it extends to the party committee government at the prefecture level and the county-level party committee government with outstanding problems. The content of the inspection focuses on understanding the provincial party committee and government to operate the national environmental protection decision-making department, solve outstanding environmental problems, implement the responsibility of environmental protection, promote the ecological civilization construction and environmental protection in the inspected areas, and promote green development. In the mode of supervision, the provincial level mainly includes convening meetings, talking with the leaders of the four groups of provincial leaders and the main responsible persons of the relevant departments, consulting documents, and visiting relevant departments. The inspections of Province and city mainly include inspection materials, on-site inspections, enterprises, investigation and evidence collection issues. The inspector group arranges special personnel, telephones and mailboxes to accept letters from the masses to send telecommunications and to handle the local deadlines, and the results are open to the public.
② China’s ecological civilization system: Faced with the grim situation of tight resource constraints, serious environmental pollution, and degraded ecosystems, we must establish an ecological civilization concept that respects nature, conforms to nature, and protects nature, and follows the path of sustainable development. In fact, the construction of ecological civilization is to raise the sustainable development to the height of green development, and to “bring trees” for future generations, that is, to leave regrets for future generations and leave more ecological assets.
③ Campaign-style governance: Campaign-style governance is motivated by the political subjects who possess certain political powers to maintain social stability and due order by political power. Through political mobilization, the enthusiasm and creativity of the class, the group and other members of society are mobilized from top to bottom. Some unexpected events or major domestic, long-delayed social problems are governed by an organized, purposeful, and large-scale mass participation process. This type of current Chinese Campaign-style governance is characterized by authority, effectiveness, sportiness, rebound and mobilization.
④ Central environmental protection inspections “Looking back”: “Looking back” is one of the links of central environmental protection inspectors. “Looking back” refers to solving problems in the first round of central environmental protection inspection, and investigating and condemning formalism and bureaucratism such as “surface rectification” and “false rectification” in ecological environmental protection.

## Appendix B

**Table A1:** Statistical characteristics and comparison of variables before and after the implementation of central environmental protection inspections.

Variable Name	Full Sample	Minimum	Maximum	Before	After	*t*-Test
AQI	79.02(54.30)	10	500	83.77(56.34)	75.49(52.47)	−8.28 ***
PM10	76.43(65.97)	0	1199	81.74(73.58)	72.48(59.39)	−9.26 ***
PM2.5	44.61(45.80)	0	493	48.27(46.10)	41.90(45.39)	−6.36 ***
CO	15.93(21.10)	0.2	134	22.45(25.31)	11.10(15.67)	−11.35 ***
NO_2_	24.62(27.03)	0.31	169	19.77(24.60)	28.22(28.18)	8.45 ***
SO_2_	17.58(22.51)	1	344	21.40(30.59)	14.73(14.24)	−6.67 ***
O_3_	78.43(44.85)	3	303	75.15(47.36)	80.87(42.73)	5.71 ***
Rain	0.36(0.48)	0	1	0.37(0.48)	0.35(0.48)	0.02
Snow	0.03(0.18)	0	1	0.05(0.21)	0.03(0.16)	−0.02 ***
Daily maximum temperature	22.59(10.17)	−11	41	20.15(10.36)	24.40(9.63)	4.25 ***
Daily minimum temperature	13.56(10.27)	−21	31	10.86(10.39)	15.57(9.72)	4.71 ***

Note: *** Denotes *p* < 0.01. The regression also includes weather variables, regional fixed effects, and time-fixed effects, such as year, month, week, and holiday. The value in () represents the heteroscedastic robust standard error of the regression coefficient.

**Table A2:** Differences in air quality control capabilities among provinces.

Province	Date	AQI	PM_2.5_	PM_10_	CO	NO_2_	SO_2_	O_3_
Hebei	31 December 2015	−11.052	−2.322	8.963	0.599	45.106 *	56.995 **	18.938 *
Yunnan	15 July 2016	−8.310	−2.250	−2.422	0.077	−3.174	2.481	−31.459 *
Neimenggu	14 July 2016	−5.580	−10.771	−16.101	0.014	5.219	−0.427	−1.204
Heilongjiang	19 July 2016	3.092	3.192	1.949	0.078	1.285	−2.388 **	14.286
Jiangsu	15 July 2016	−9.743	−5.976	−8.249	0.106	−2.956	2.988	15.798
Jiangxi	14 July 2016	−1.961	−0.372	4.384	−0.072	3.969	6.902 *	−0.373
Henan	16 July 2016	21.116	−6.696	−12.034	−0.161	−8.324	−4.719	−3.538
Guangxi	14 July 2016	−7.183	−12.352 **	−18.107 *	0.014	−8.697 ***	−1.362	−17.358 *
Ningxia	12 July 2016	11.527	−16.602 *	−28.234	−0.007	0.833	−8.757 **	9.218
Beijing	29 November 2016	−67.787	−65.099	−74.229	−0.824	−6.809	0.471	5.714
Shanghai	28 November 2016	−4.728	−11.703	10.553	−0.338 *	4.616	−3.577	23.911
Hubei	26 November 2016	43.017	29.954	47.209	−0.352	7.662	0.762	19.272
Guangdong	28 November 2016	2.874	−12.233	−6.160	−0.245 *	−23.391 *	−0.730	−8.262
Chongqing	24 November 2016	−0.804	3.177	3.602	−0.106	−7.348	−4.479 *	9.497
Shanxi	28 November 2016	−30.753	−27.880	−56.723	−0.452	−14.723	3.166	7.285
Gansu	30 November 2016	−19.262	1.342	−47.893	0.469 *	19.656 **	−7.580	−0.697
Hunan	25 April 2017	−13.49 ***	−8.639 **	−19.631 ***	−8.228 **	−0.231	−0.265	2.759
Guizhou	26 April 2017	−9.861 *	−4.250	−10.779 *	1.618	0.072 *	2.316	−17.896***
Liaoning	25 April 2017	−10.770	−17.018 *	−52.632 ***	0.189	−0.037	4.424	−23.116 **
Shanxi	28 April 2017	−11.338	−6.227	−44.148*	−3.115	0.040	11.667	17.865 *
Tianjin	28 April 2017	−26.502	−8.776	−67.681 ***	−9.054 *	0.150	−5.17 *	5.875
Fujian	24 April 2017	−12.237	−5.909	−19.955 *	−5.932	0.319	−0.409	−5.315
Anhui	27 April 2017	−16.540	−3.135	−19.507	−10.157 *	−0.083	0.349	24.409 **
Jilin	11 August 2017	−12.445	1.015	−3.344	0.065	−3.708	1.736	−24.031 *
Shandong	10 August 2017	−32.350 *	−10.848	−31.146	−0.284	−2.694	−3.556 *	25.682
Hainan	10 August 2017	−11.162 *	−5.689 **	−11.163 ***	−0.138 ***	−9.945 ***	−1.702 ***	−21.679 *
Xizang	15 August 2017	−9.369	−0.962	−2.813	−0.098 ***	−2.295	0.630	−16.807
Qinghai	8 August 2017	4.483	4.779	3.025	0.001	−16.40 ***	−3.560	18.737
Xinjiang	11 August 2017	6.963	−2.120	−14.851	−0.110	−2.650	0.072	1.413
Sichuan	7 August 2017	−22.444 *	3.874	5.665	0.059	−3.488	1.652 **	−25.466
Zhejiang	11 August 2017	−16.459	−16.263 *	−17.041 *	−0.133	−2.101	−1.532	−17.835

Note: * Denotes *p* < 0.10. ** Denotes *p* < 0.05. *** Denotes *p* < 0.01. The regression also includes weather variables, regional fixed effects, and time-fixed effects, such as year, month, week, and holiday.
